# Syntactic analysis of SMOSS model combined with improved LSTM model: Taking English writing teaching as an example

**DOI:** 10.1371/journal.pone.0312049

**Published:** 2024-11-15

**Authors:** Ke Yan

**Affiliations:** Department of Public Instruction, Nanyang Medical College, Nanyang, Henan, China; The University of Lahore, PAKISTAN

## Abstract

This paper explores the method of combining Sequential Matching on Sliding Window Sequences (SMOSS) model with improved Long Short-Term Memory (LSTM) model in English writing teaching to improve learners’ syntactic understanding and writing ability, thus effectively improving the quality of English writing teaching. Firstly, this paper analyzes the structure of SMOSS model. Secondly, this paper optimizes the traditional LSTM model by using Connectist Temporal Classification (CTC), and proposes an English text error detection model. Meanwhile, this paper combines the SMOSS model with the optimized LSTM model to form a comprehensive syntactic analysis framework, and designs and implements the structure and code of the framework. Finally, on the one hand, the semantic disambiguation performance of the model is tested by using SemCor data set. On the other hand, taking English writing teaching as an example, the proposed method is further verified by designing a comparative experiment in groups. The results show that: (1) From the experimental data of word sense disambiguation, the accuracy of the SMOSS-LSTM model proposed in this paper is the lowest when the context range is "3+3", then it rises in turn at "5+5" and "7+7", reaches the highest at "7+7", and then begins to decrease at "10+10"; (2) Compared with the control group, the accuracy of syntactic analysis in the experimental group reached 89.5%, while that in the control group was only 73.2%. (3) In the aspect of English text error detection, the detection accuracy of the proposed model in the experimental group is as high as 94.8%, which is significantly better than the traditional SMOSS-based text error detection method, and its accuracy is only 68.3%. (4) Compared with other existing researches, although it is slightly inferior to Bidirectional Encoder Representations from Transformers (BERT) in word sense disambiguation, this proposed model performs well in syntactic analysis and English text error detection, and its comprehensive performance is excellent. This paper verifies the effectiveness and practicability of applying SMOSS model and improved LSTM model to the syntactic analysis task in English writing teaching, and provides new ideas and methods for the application of syntactic analysis in English teaching.

## Introduction

In English writing teaching, syntactic analysis has always been a key step for learners to understand and use grammatical rules, which is very important for improving English writing ability [1]. Syntactic analysis can not only help learners to identify the grammatical relationship between words and phrases in sentences, but also clarify the structure of sentences, so that learners can better understand grammatical rules, and then improve the accuracy of writing and fluency of expression. However, in the face of complex syntactic structure and complicated grammatical rules, many learners are often confused, which in turn affects the writing quality [2]. Therefore, it is of great educational significance and practical value to develop an effective syntactic analysis method to help learners improve their syntactic understanding and writing ability.

In recent years, with the development of natural language processing technology, syntactic analysis methods have been significantly improved. Traditional rule-based methods are gradually replaced by methods based on machine learning and deep learning, especially the deep learning model, which performs well in natural language processing tasks, is widely used. However, the current research mainly focuses on how to improve the effect of syntactic analysis through a single model, and there is still a lack of exploration on the application of different model combinations in specific educational scenes.

The motivation of this paper is to explore how to combine sequential matching on sliding window sequences (SMOSS) model with improved Long Short-Term Memory (LSTM) model and apply it to the task of syntactic analysis in English writing teaching to make up for the shortcomings of existing methods in educational scenes. SMOSS model captures the local features of sentences by sliding windows, which can effectively extract syntactic information and provide support for syntactic analysis [3, 4]. As a kind of recurrent neural network (RNN) with memory unit, LSTM model performs well in sequence modelling tasks, especially in dealing with long sequence dependence in natural language tasks [1, 5, 6].

In this paper, firstly, the structure of SMOSS model is deeply analyzed, and the traditional LSTM model is optimized by introducing Connectist Temporal Classification (CTC), thus an error detection model suitable for English texts is proposed. By combining the SMOSS model with the optimized LSTM model, a comprehensive syntactic analysis framework is constructed, and the structure and code of the framework are designed and implemented. In the experimental part, the semantic disambiguation performance of the model is tested by using SemCor dataset, and then the application effect of the proposed method in the actual English writing teaching scene is verified by group comparison experiments.

Although the Transformer model has made remarkable progress in the field of natural language processing in recent years, the LSTM model still shows higher efficiency in some sequential tasks. Specifically, the LSTM model is excellent in dealing with tasks with long sequence dependencies, especially those that need to capture the time sequence and causality in the input sequence. Therefore, the improved LSTM model is still a reliable and effective choice in the face of tasks such as English syntactic analysis and text error detection. The contribution of this paper is to combine the SMOSS model with the improved LSTM model, put forward a new syntactic analysis method, and verify its application effect in English writing teaching. Through this exploratory research, this paper provides new ideas and methods for the further study of syntactic analysis tasks.

## Literature review

SMOSS model is a method based on sliding window sequence matching [7]. In recent years, with the development of deep learning technology, more and more researches began to focus on combining different models to improve the performance of natural language processing tasks. For example, Yan proposed a comprehensive syntactic analysis framework by combining the SMOSS model and the improved LSTM model, which performed well in the task of syntactic analysis in English writing teaching [8]. The study not only optimized the LSTM model, but also improved the error detection ability of the model by introducing the connection time series classification method. It significantly improved the accuracy of word sense disambiguation and syntactic analysis. This showed that the optimization and combination of the model can significantly improve the effect of the model in specific language tasks. In the field of natural language processing, LSTM model performs well in language modelling, machine translation, semantic analysis and other tasks. In the task of emotion classification, the combination of syntax and semantic analysis has also been effectively applied. Zhang et al. proposed a model combining syntactic and semantic analysis in aspect-level emotion classification, which integrated aspect information and context information through an effective fusion mechanism, thus improving the classification performance of the model [9]. The success of this study further showed that in natural language processing, combining multiple analysis methods can make up for the shortcomings of a single method and improve the overall performance of the task. In addition, the research in the field of text classification also proved the advantages of the hybrid model. Jang et al., using the mixed model of Bi-LSTM and Convolutional Neural Networks (CNN), combined with attention mechanism, can better capture long sequence dependencies and extract advanced features, thus improving the accuracy of text classification and F1 score [10]. This study showed that by combining LSTM, CNN and attention mechanism, the performance of deep learning model in natural language processing tasks can be further optimized, especially in complex tasks that need to remember a lot of features. Meshram and Anand Kumar proposed a deep context semantic text similarity model based on LSTM networks. By introducing the deep context mechanism to collect high-level semantic information, the model was applied to multiple data sets, and the effectiveness of the model was verified. The research showed that when dealing with semantic similarity tasks, LSTM network combined with deep context embedding not only achieved excellent performance in regression and classification tasks, but also exceeded the benchmark of human labelling. This discovery further proved the potential of LSTM in capturing semantic similarity between sentences, especially in tasks involving deep semantic knowledge extraction [11].

At present, although the application of SMOSS model and LSTM model in English writing teaching is still in the primary stage, some experimental studies have made some progress. Applying SMOSS model and LSTM model to the task of syntactic analysis in English writing teaching is expected to improve the accuracy and practicability of syntactic analysis. However, there are still some challenges, such as the complexity of the model, the matching of data sets and the handling of error types. Therefore, it is necessary to further study and explore the optimization and improvement of SMOSS model and LSTM model in English writing teaching. Based on the literature review, this paper will continue the exploration of SMOSS model and LSTM model in English writing teaching.

## Research on syntactic analysis model

### Structural analysis of SMOSS model

The core idea of SMOSS model is to extract local features from sentences by sliding windows to capture local semantic information of sentences [12, 13]. Its structure consists of the following key components: input layer, sliding window, feature extractor, feature matching and output layer [14]. Among them, the core of SMOSS model is matching function, which performs matching operation on sliding window. Assuming that the sliding window size is *w* and the sliding window position is *p*, the matching function *M*(*p*) can be expressed as Eq (1) [15].

M(p)=f(X[p:p+w]) (1)

where *X*[*p*: *p*+*w*] denotes a word-oriented quantum sequence in a window of length *w* starting from position *p*. The function *f* (·) is a nonlinear mapping function, which is used to map the words in the window to the quantum sequence to the matching representation space.

In order to capture the relationship between matching representations in different positions, SMOSS model introduces matching attention mechanism. Assuming that the attention weight between matching representations is *A*, when the *i*-th matching representation *M*(*p*_*i*_) is considered, its weighted representation is Eq (2) [16].

$$M_{\mathrm{weighted}}(p_{i})=\sum_{j=1}^{N}A_{ij}\cdot M(p_{j})$$ (2)

where *A*_*ij*_ denotes the attention weight between the *i*-th matching representation and the *j*-th matching representation.

The calculation of attention mechanism is shown in Eq (3):

$$\mathrm{Attention}\;\mathrm{Weight}(\alpha_{i})=softmax(W_{a}\cdot tanh(W_{m}\cdot M(p)+b_{m})+b_{a})$$ (3)

A new parameter matrix *W*_*a*_ and *W*_*m*_ and the corresponding bias items *b*_*m*_ and *b*_*a*_ are introduced for learning attention weights.

Input the weighted representation of the matching representation into a fully connected layer to obtain the overall SMOSS representation, as shown in Eq (4) [17].

$$SMOSS_{\mathrm{output}}=ReLU(W\cdot M_{\mathrm{weighted}}+b)$$ (4)

where *W* and *b* denote the parameters of the fully connected layer, and ReLU represents the activation function.

The input layer of SMOSS model is responsible for receiving the text data to be processed. In the task of parsing, the input layer transforms English sentences into word sequences and embeds them, that is, each word is mapped into a dense vector representation. Such an embedded representation can better represent the semantic relationship between words. Sliding window is one of the core components of SMOSS model. It cuts the input word sequence into several sub-sequences with fixed length, and then performs feature extraction and matching on each sub-sequence [18]. The size of sliding window is an important super parameter, which determines the range of local features.

### Optimization of improved LSTM model based on CTC

In the improved LSTM model, CTC loss function is introduced. This innovative improvement enables the model to better learn the internal patterns and characteristics of sequence data during the training process. The use of CTC loss function eliminates the need of traditional manual feature engineering and realizes end-to-end sequence learning, so that the model can learn a higher-level representation directly from the original data without relying on artificially designed features. This end-to-end learning method not only improves the adaptability of the model, but also enhances the generalization ability of the model, so that it can better adapt to the characteristics of different tasks and data sets. In the task of sequence labelling, the improved LSTM model with CTC loss function shows higher performance, because it can better capture the complex patterns and dependencies in sequence data, thus improving the accuracy and efficiency of the model. This innovative improvement not only improves the performance of the model, but also provides a more concise and effective method for solving the sequence labelling task.

CTC model is an end-to-end model for sequence learning, which is mainly used to process sequence data with indefinite output sequences. Its basic structure consists of the following key components:

1. Input sequence and output sequence: CTC model accepts input sequence and output sequence as training data. The input sequence is usually a time sequence, such as the time step of speech signal or text data. The output sequence is a tag sequence corresponding to the input sequence, which can be a sequence with indefinite length.
2. Tag set: The output layer of CTC model is usually associated with the tag set, which defines the possible output categories. In the training process, CTC model needs to learn to map the input sequence to the correct tag sequence.
3. Blank mark: CTC model introduces a special mark called blank mark, which is used to represent the blank part in the input sequence. The introduction of Blank mark enables CTC model to deal with the inconsistency between the input sequence and the output sequence.
4. Network structure: CTC model usually adopts neural network structure, such as RNN or LSTM, as its basic framework. These networks can process the input sequence step by step and generate the corresponding output sequence.
5. CTC loss function: CTC model uses CTC loss function as the optimization goal of training. CTC loss function considers all possible output sequences, and guides the updating of model parameters by calculating the differences between these sequences and real tag sequences.
6. Decoding algorithm: In the prediction stage, CTC model usually uses decoding algorithm to decode the output sequence to obtain the final prediction result. The decoding algorithm can search and correct the tag sequence according to the probability distribution of the model output, thus improving the accuracy of prediction.

In this paper, CTC is applied to the task of syntactic analysis, and the LSTM model is optimized by CTC loss function to improve the syntactic analysis effect in English writing teaching [19].

In the sequence labelling task, given the input sequence $X=(x_{1},x_{2},\ldots ,x_{T})$, it is necessary to predict the output sequence $Y=(y_{1},y_{2},\ldots ,y_{T})$. *T* represents the length of the input sequence *X* and the length of the output sequence *Y*. However, because the sequence length of different samples may be different, the traditional labelling data usually need to be strictly aligned, that is, the input sequence and the output sequence are required to have the same length [20]. This will be a challenge for the task of syntactic analysis, because learners’ sentence structures are diverse, resulting in different sentences with different lengths.

The key of CTC principle is to solve the sequence alignment problem by defining blank symbol Ø and repeated symbol *r*. Assuming that the input sequence is "hello", the possible output sequences are "healo", "helo" or "hello". The goal of CTC model is to find all possible alignments and calculate the probability of each alignment. In order to achieve this, CTC introduces blank symbols in the output sequence *Y*, indicating that there may be silent blank parts, such as "heØaØllo". The repetition symbol *r* is used to represent the same characters in succession, such as "heØarØllo". By introducing blank symbols and repeated symbols, CTC can find all possible alignment paths [21].

When calculating the CTC loss function, it is necessary to accumulate all possible alignment paths to get the difference between the output sequence *Y* and the real output sequence [22]. Minimizing the loss function will make the model better adapt to the mapping relationship between input sequence and output sequence, thus optimizing the whole LSTM model.

Given the input sequence $X=(x_{1},x_{2},\ldots ,x_{T})$ and the output sequence $Y=(y_{1},y_{2},\ldots ,y_{T})$, the CTC loss function is defined as Eq (5).

$$L_{CTC}=-log\sum_{\pi \in B^{-1}(Y)}P(\pi |X)$$ (5)

Where *B*^−1^(*Y*) denotes the set of all possible alignment paths of the output sequence *Y*. *P*(*π*|*X*) represents the conditional probability of output sequence *π* given input sequence *X*. The goal of the loss function is to minimize *L*_*CTC*_ to optimize the model parameters and make the difference between the predicted output sequence and the real output sequence as small as possible.

By introducing blank symbols and repeated symbols, CTC’s flexible sequence alignment mechanism enables the improved LSTM model to learn without complete alignment labels and better adapt to the diverse sentence structures in learners’ writing expressions. This provides new ideas and methods for the application of syntactic analysis in English writing teaching, and lays the foundation for the innovation of this study.

In order to apply CTC method to improve the optimization of LSTM model, the CTC loss function is connected with the output layer of LSTM. In the traditional LSTM model, the output layer usually maps the hidden state of LSTM to the classification label space through the fully connected layer. However, in this paper, the output sequence of LSTM model is directly used as the input sequence of CTC without introducing additional full connection layer.

In LSTM model, the hidden state sequence $H=(h_{1},h_{2},\ldots ,h_{T})$ is obtained by recursive calculation. *h*_*T*_ represents the hidden state at time *T*, and the output sequence $O=(o_{1},o_{2},\ldots ,o_{T})$ of LSTM model. The traditional LSTM model will use the fully connected layer to map the hidden state to the target label space, and then carry out the classification task. However, in this paper, the output sequence of LSTM model is directly used as the input sequence of CTC without introducing additional full connection layers.

The combination mode of the improved LSTM model based on CTC is shown in Table 1 [23]:

**Table 1:** Combination steps of improved LSTM model based on CTC.

Step number	Specific content
**1**	Input the input sequence *X* into the LSTM model, and get the hidden state sequence $H=(h_{1},h_{2},\ldots ,h_{T})$ through recursive calculation.
**2**	The output sequence $O=(o_{1},o_{2},\ldots ,o_{T})$ of the LSTM model is associated with the hidden state *H*, and the output sequence *O* is taken as the input sequence of CTC.
**3**	CTC model uses blank symbol Ø and repeated symbol *r* to find all possible alignment paths and learn the corresponding relationship between output sequences and real labels.
**4**	Minimize the CTC loss function to optimize the parameters of the whole model, that is, ${min}_{\theta }L_{CTC}(\theta )$, where *θ* represents the parameters of the model.

The goal of the training process of the improved LSTM model based on CTC is to optimize the model parameters by minimizing the CTC loss function to achieve better syntactic analysis effect. In the training process, the whole model is jointly trained by using back propagation algorithm combined with CTC loss function. Let *P*(*Y*|*X*) be the probability between the output sequence o of CTC model and the real output sequence *Y*, and the CTC loss function is defined as $L_{CTC}=-logP(Y|X)$. The parameters of the whole model are optimized by minimizing CTC loss.

In the training process, the Stochastic Gradient Descent (SGD) algorithm is used for optimization, and the learning rate adjustment strategy is used to speed up the convergence of the model. In addition, in order to prevent over-fitting, techniques such as Dropout and L2 regularization are introduced. Specifically, for each sample (X, Y), the gradient $\nabla_{\theta }L_{CTC}(X,Y;\theta )$ of its CTC loss function with respect to parameter *θ* is calculated. Then, the learning rate *α* is used to update the parameters, and the updating formula is shown in Eq (6) [24].

$$\theta \leftarrow \theta -\alpha \cdot \nabla_{\theta }L_{CTC}(X,Y;\theta )$$ (6)

where *α* denotes the learning rate, which is used to control the pace of parameter update.

In recent years, many advanced prediction models, such as DeepAVP-TPPred [25], iFPS-MV-BitCN [26], Deepstacked-AVPs [27], AIPs-SnTCN [28], StackedEnC-AOP [29] and pAtbP-EnC [30], have been widely used in various natural language processing tasks. These models are usually combined with feature extraction technology of specific tasks through multi-layer neural network architecture, which significantly improves the prediction accuracy and generalization ability of the models. The improved LSTM+CTC model proposed in this paper draws lessons from the successful experience of these advanced models in dealing with complex sequence tasks, and innovatively designs syntactic analysis in English writing teaching.

In order to evaluate the generalization of the model, the cross-validation method is adopted in the training process, and the data set is divided into multiple folds, and each fold is trained and verified, so as to obtain a more robust model performance evaluation. In addition, Early Stopping is introduced to prevent the model from over-fitting in the training process, that is, when the performance of the verification set is no longer improved, the training is terminated in advance. Combined with these methods, the generalization and stability of the model are effectively evaluated.

In order to prevent the model from over-fitting, Dropout technology is introduced, which can randomly set the output of some neurons to zero, thus reducing the dependence between neurons and enhancing the generalization ability of the model [31]. Meanwhile, L2 regularization is also adopted, and the L2 norm of parameters is introduced into the loss function to suppress the situation that the parameters are too large, thus further preventing the model from over-fitting the training data [32]. Dropout technology reduces the dependence between neurons and enhances the generalization ability of the model by randomly setting the output of some neurons to zero. Let *h*_*i*_ represent the output of the *i*th neuron, and in the training process, a retention probability *p* is used to control the retention and discarding of neurons, that is, Eq (7):

$$h_{i}=\left\{ \begin{array}{l} h_{i}\cdot r_{i},\;with\;probability\;p\\ 0,\;with\;probability\;1-p \end{array}\right.$$ (7)

where *r*_*i*_ denotes a random number that obeys the uniform distribution *U* (0,1). During the test, the output of all neurons is kept instead of using Dropout.

L2 regularization suppresses the condition that the parameters are too large by introducing L2 norm into the loss function, thus further preventing the model from over-fitting the training data. The calculation process is shown in Eq (8).

$$R(\theta )=\sum_{i=1}^{N}\theta_{i}^{2}$$ (8)

where *θ* denotes the model parameter. *N* is the total number of model parameters. The L2 regularization term is added to the original CTC loss function to obtain the regularized loss function, as shown in Eq (9).

$$L_{reg}=L_{CTC}+\lambda \cdot R(\theta )$$ (9)

where *λ* denotes a regularization parameter used to control the regularization intensity.

During the training of the LSTM model, the key parameters in Table 2 are used:

**Table 2:** Key parameters of LSTM training model.

Parameter name	Scale	Selection basis
**LearningRate**	0.001–0.01	Through cross-validation selection, the model convergence speed is ensured to be moderate.
**HiddenUnits**	128,256	Ensure that the model has sufficient representation ability and avoid over-fitting.
**BatchSize**	32,64	Balancing computational efficiency and model performance
**Dropout probability**	0.2–0.5	Prevent the over-fitting of the model and enhance the generalization ability of the model.
**L2 regularization coefficient**	0.001–0.01	Control the size of model parameters to prevent over-fitting.

The selection of the above parameters is based on the performance on the verification set, and the performance and stability of the model are ensured through the parameter adjustment experiment.

### Design and implementation of comprehensive syntactic analysis framework

**Fig 1:**
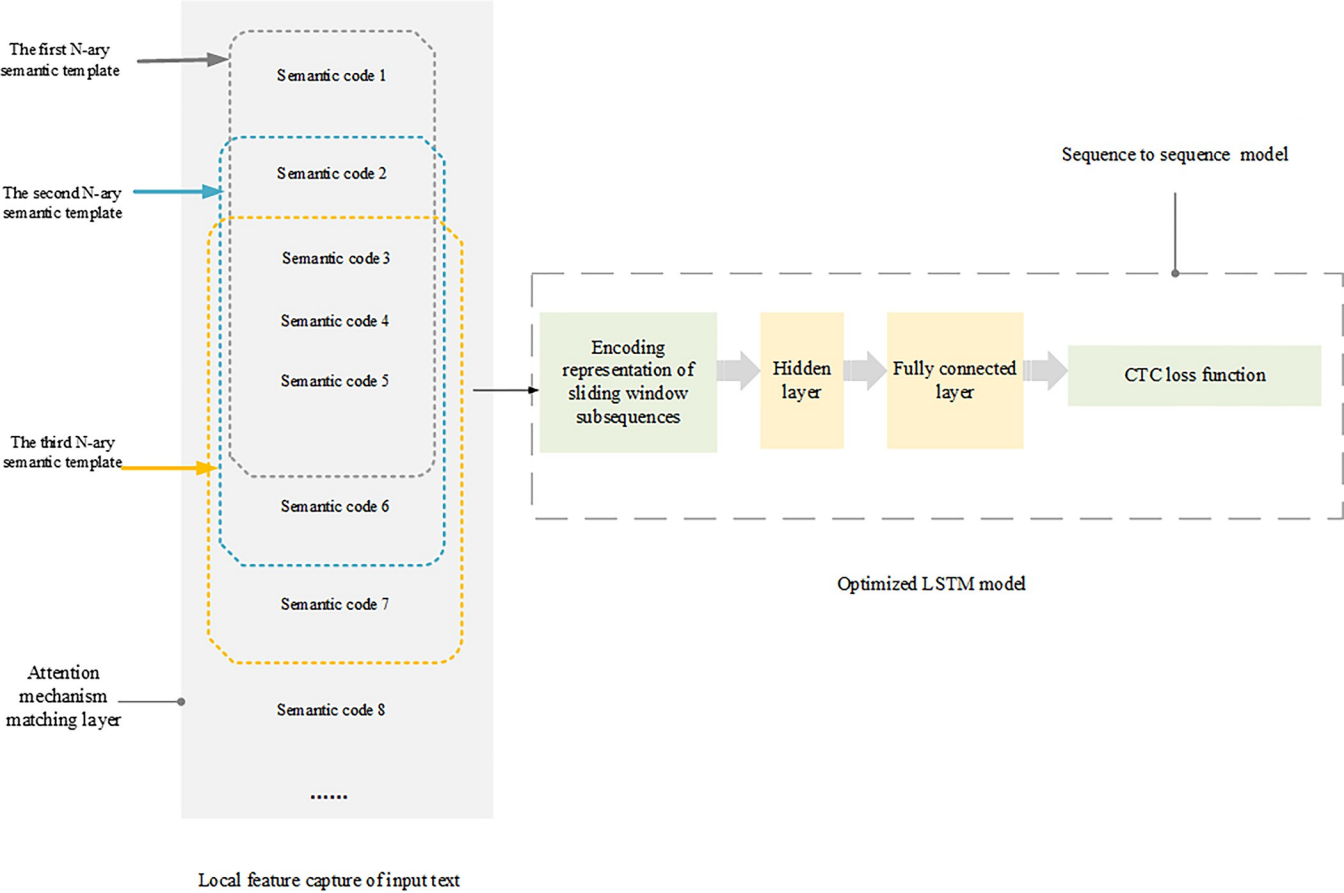
Overall model framework.

In the framework of comprehensive syntactic analysis, the SMOSS model is integrated with the optimized LSTM model. Specifically, firstly, the SMOSS model is used to capture the local features of the input text and obtain the coded representation of the sliding window subsequence. Then, these encoded representations are input into the optimized LSTM model, and the encoded representation of the whole text is obtained by LSTM encoder. The overall model framework is shown in Fig 1:

In the framework of comprehensive syntactic analysis, attention mechanism plays a crucial role. By calculating the similarity between each sliding window subsequence and other subsequence, attention mechanism can effectively capture the interaction between local features and global features. This mechanism enables the model to focus on the most relevant information when dealing with complex syntactic structures, thus improving the accuracy of analysis.

For the construction of seq2seq model, the encoder uses the improved LSTM model to encode the input text into a fixed-length context vector *C*. The decoder relies on this context vector to generate the output sequence step by step. In this process, the decoder not only relies on the output of the previous step, but also continuously refers to the key information in the input sequence through the attention mechanism, thus ensuring the semantic and structural consistency between the generated text and the input text. This design enables the seq2seq model to maintain high accuracy and fluency when dealing with sequence generation tasks.

In order to make better use of the context information between SMOSS model and LSTM model, a matching layer is introduced into the framework. The matching layer adopts attention mechanism, and realizes the interaction between local features and global features by calculating the similarity between each sliding window subsequence and other subsequence. Specifically, assuming that there are *N* sliding window subsequence in the input text, the output of the matching layer can be expressed as Eq (10).

$$Attention(Q,K,V)=softmax(\frac{QK^{T}}{\sqrt{d_{k}}})V$$ (10)

where *Q*, *K*, *V* denote the local features of SMOSS model output, the global features of LSTM model output and the features that need to be fused, and *d*_*k*_ is the feature dimension.

In order to better cope with the task of error detection in English writing teaching, the LSTM model is extended and a sequence-to-sequence (seq2seq) model is constructed. The model includes an encoder and a decoder, which are used to encode the input text and generate the corrected text respectively.

In the encoder part, the improved LSTM model is used to encode the input text, and the coding representation *C* is obtained. In the decoder part, another LSTM model is used to generate the corrected text step by step. Assuming that the target text is *Y*, the goal of the decoder is to maximize the conditional probability of generating the target text, that is, Eq (11).

$$P(Y|X)=\prod_{t=1}^{T}P(y_{t}|y_{1},y_{2},\ldots ,y_{t-1},C)$$ (11)

where *T* denotes the target text length.

In the implementation of the comprehensive parsing framework, the SMOSS model and the improved LSTM model are realized by using Python programming language and TensorFlow, a deep learning framework. Batch training and optimizer are used to train the model, and cross entropy loss function is used to measure the difference between the prediction results of the model and the real label.

## Analysis of the evaluation results of syntactic analysis model effect

### Dataset introduction and experimental environment

Firstly, the SemCor data set is selected as a main evaluation data set in this study [33]. SemCor is a semantic contextual tagging corpus created by Princeton University, which contains a large number of semantic tagging of English sentences and words. This data set is widely used in natural language processing tasks such as word sense disambiguation and syntactic analysis. This article uses 1,000 data in the SemCor dataset.

In addition to SemCor data set, an English writing teaching data set is also built to be closer to the actual scene of English writing teaching. This data set contains sentences and paragraphs written by learners in English writing teaching, including examples with different grammatical difficulty and writing level. The sentences in the data set include all kinds of grammatical errors, such as inconsistent subject and predicate, tense errors, improper use of articles and so on. A total of 500 self-built datasets are collected.

The experiment is conducted on a personal computer equipped with Intel Core i7 processor and 16GB memory. The deep learning framework used is TensorFlow 2.5.0, and the model training is accelerated by GPU.

Three independent experiments are carried out to ensure the stability and repeatability of the results. The number of iterations in each experiment is 100 epochs, and the batch size of each epoch is 64. Adam optimizer is adopted, and the learning rate is set to 0.001, β1 to 0.9 and β2 to 0.999. In the process of model training, the early stop strategy is adopted. When the loss on the verification set has not decreased for 10 consecutive epochs, the training is stopped to prevent over-fitting.

The differences between the three experiments are mainly reflected in the following aspects: First, in each experiment, different random seeds are used to divide the training set and the test set to ensure different sample distribution. Secondly, in Experiment 2, the number of hidden layer units of LSTM model is fine-tuned from 128 to 256 to observe the influence of model capacity on the results. Finally, in Experiment 3, data enhancement technology is introduced to improve the generalization ability of the model by generating more training samples. All these adjustments are aimed at comprehensively evaluating the performance of the model under different conditions.

### Analysis of model performance evaluation results

The train and test set of SemCor data set are divided into 80:20, and the comprehensive syntactic analysis framework model constructed in this paper is trained and evaluated. Taking the traditional SMOSS-based text error detection method as the control group and the comprehensive syntactic analysis framework model in this paper as the experimental group, the evaluation results of the model performance are compared, and the results are shown in Table 3 for three experiments.

**Table 3:** Model performance evaluation results.

Number of experiments	Group	Accuracy	Recall rate	F1 score
**1**	Experimental group	0.890	0.870	0.882
	Control group	0.730	0.711	0.721
**2**	Experimental group	0.891	0.870	0.882
	Control group	0.731	0.712	0.723
**3**	Experimental group	0.890	0.873	0.884
	Control group	0.732	0.711	0.720

**Fig 2:**
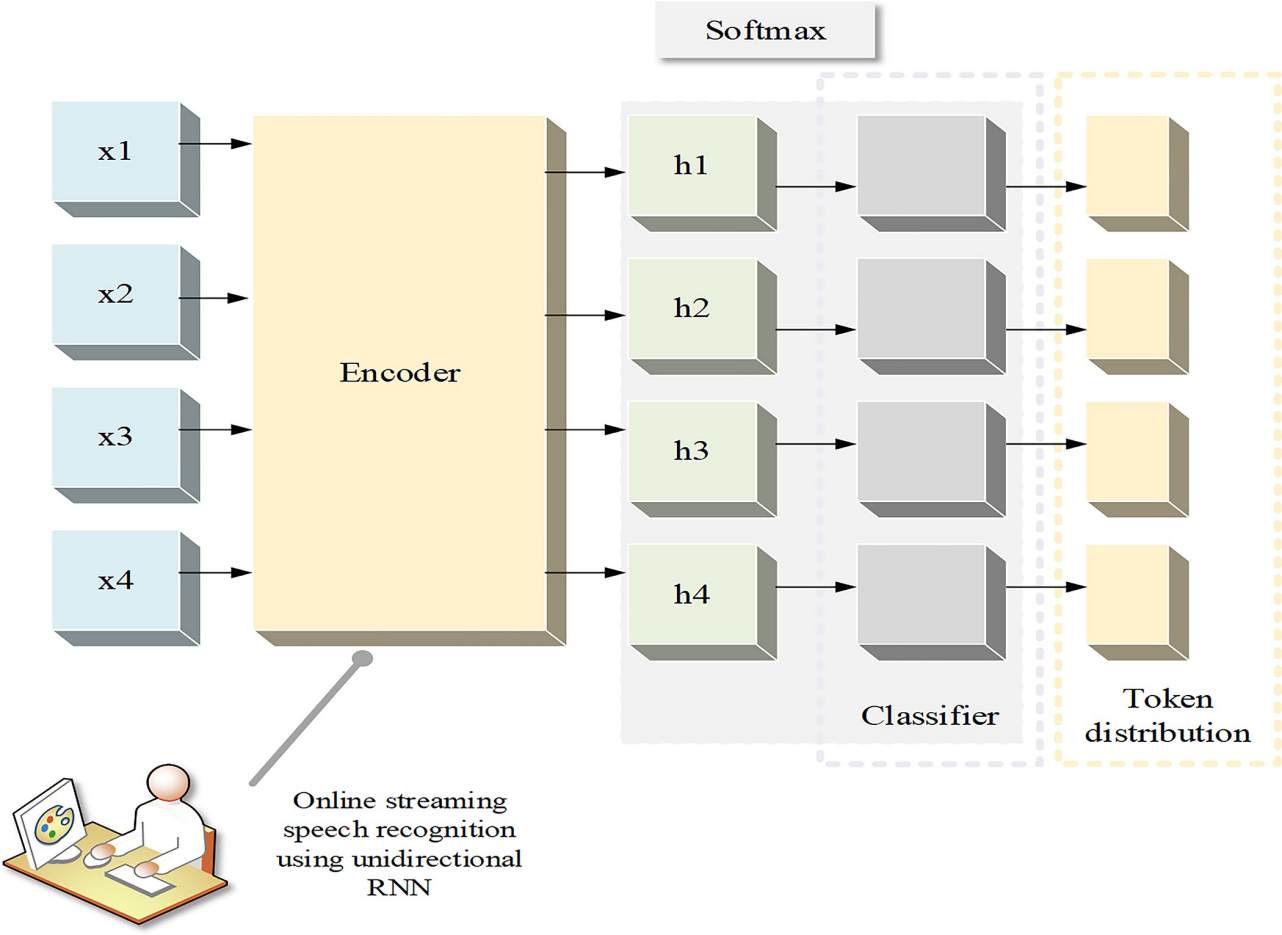
Comparison chart of model evaluation results.

The visualization result is shown in Fig 2.

Fig 2 shows that the accuracy of the experimental group is between 0.890 and 0.891, the recall rate is between 0.870 and 0.893, and the F1 score is between 0.882 and 0.884. The accuracy of the control group is 0.7030 at the minimum and 0.732 at the maximum, and the recall rate and F1 score are also around 0.710. According to these data, it can be concluded that the experimental group performs better in syntactic analysis tasks than the control group.

### Analysis of experimental results of word sense disambiguation

SemCor data set is used to test the semantic disambiguation performance of the model to evaluate the effect of the improved SMOSS-LSTM model in disambiguation. Several ambiguous words are selected as test samples, including "bank", "plant" and other common ambiguous words. By embedding these words into sentences, test data with different contexts are constructed and input into the improved SMOSS-LSTM model for disambiguation. The choice of these ambiguous words is based on their polysemy in different contexts, and these words may have multiple meanings in practical use. For example,’ bank’ can mean’ bank’ or’ river bank’, while’ plant’ can mean’ plant’ or’ factory’. By referring to the different semantic labels of these words in SemCor dataset, their polysemy is determined. The construction of test set depends on embedding these words in different contexts, thus generating test samples with different contexts to test the disambiguation ability of the model. The comparison of disambiguation accuracy between the experimental group and the control group is shown in Table 4.

**Table 4:** Experimental results of word sense disambiguation.

Number of experiments	Group	3+3	5+5	7+7	10+10
**1**	Experimental group	0.821	0.870	0.890	0.831
	Control group	0.760	0.811	0.852	0.780
**2**	Experimental group	0.792	0.850	0.881	0.812
	Control group	0.753	0.791	0.841	0.763
**3**	Experimental group	0.810	0.862	0.901	0.820
	Control group	0.741	0.780	0.851	0.772

**Fig 3:**
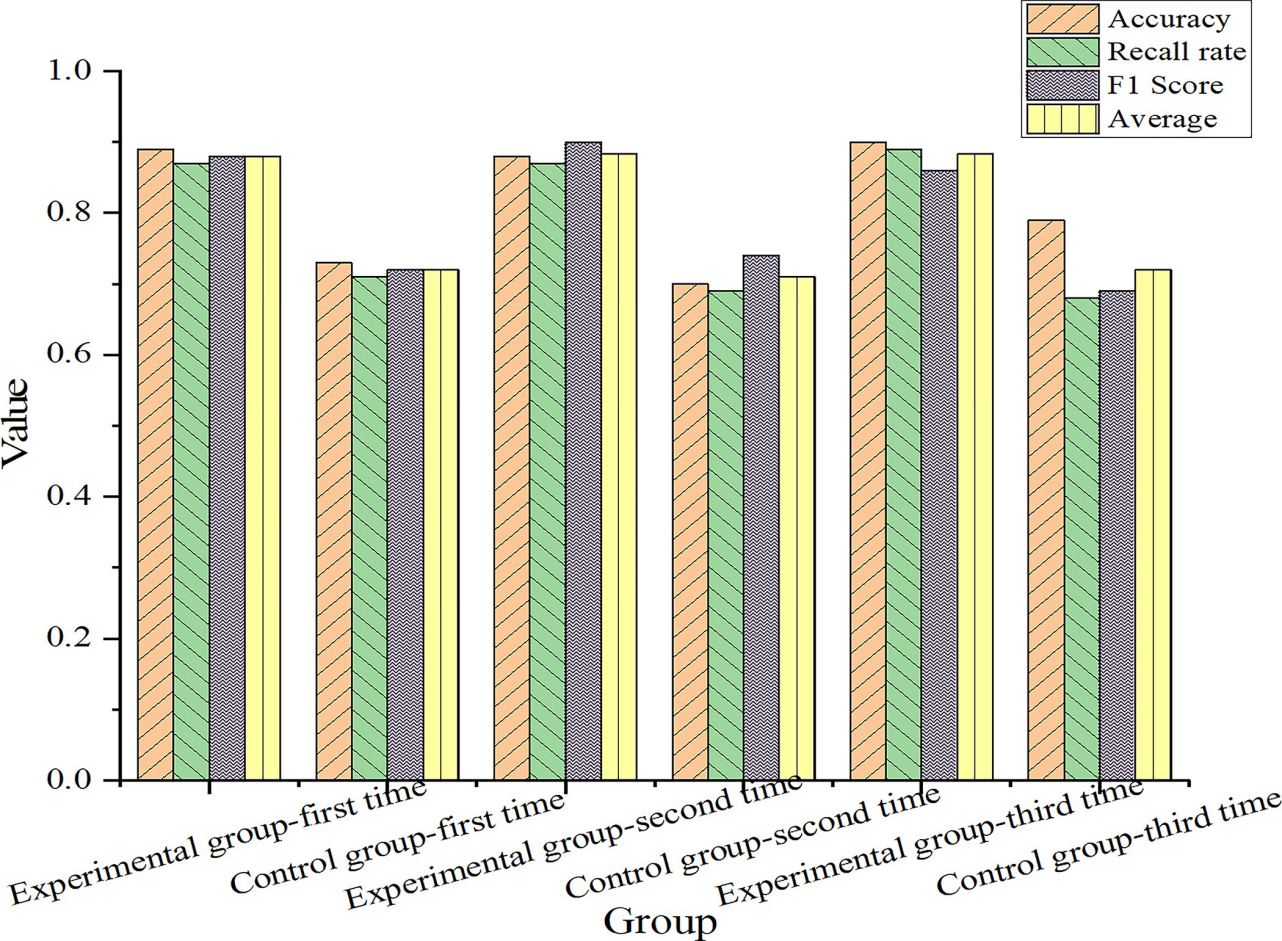
Comparison chart of word sense disambiguation results.

The diagram is drawn according to Table 4, as shown in Fig 3.

According to Fig 3, by comparing the results of the experimental group and the control group, it can be found that the improved SMOSS-LSTM model in the experimental group is superior to the benchmark model in the control group in disambiguation accuracy. The improved SMOSS-LSTM model makes better use of contextual information, especially in a larger context, and can understand sentence context more comprehensively, thus eliminating ambiguity more accurately. However, the benchmark model in the control group is not as sensitive to the use of context information as the improved SMOSS-LSTM model, resulting in low accuracy.

### Validation of syntactic analysis effect and analysis of comparative experimental results

In the verification and comparison of syntactic analysis effect, the control group is set as the traditional dependency parser. The experimental group is the improved SMOSS-LSTM model. The experiments are still carried out with sentences in SemCor data set, and different sample sizes (50, 100, 150) are selected to investigate the generalization ability and effect of the model under different data sizes. The results are shown in Table 5.

**Table 5:** Validation of syntactic analysis effect and comparison of experimental results.

Number of experiments	Group	50	100	150
**1**	Comprehensive analysis framework	0.850	0.895	0.821
	Traditional dependency parser	0.711	0.741	0.721
**2**	Comprehensive analysis framework	0.860	0.882	0.820
	Traditional dependency parser	0.732	0.711	0.722
**3**	Comprehensive analysis framework	0.870	0.891	0.840
	Traditional dependency parser	0.732	0.720	0.712

**Fig 4:**
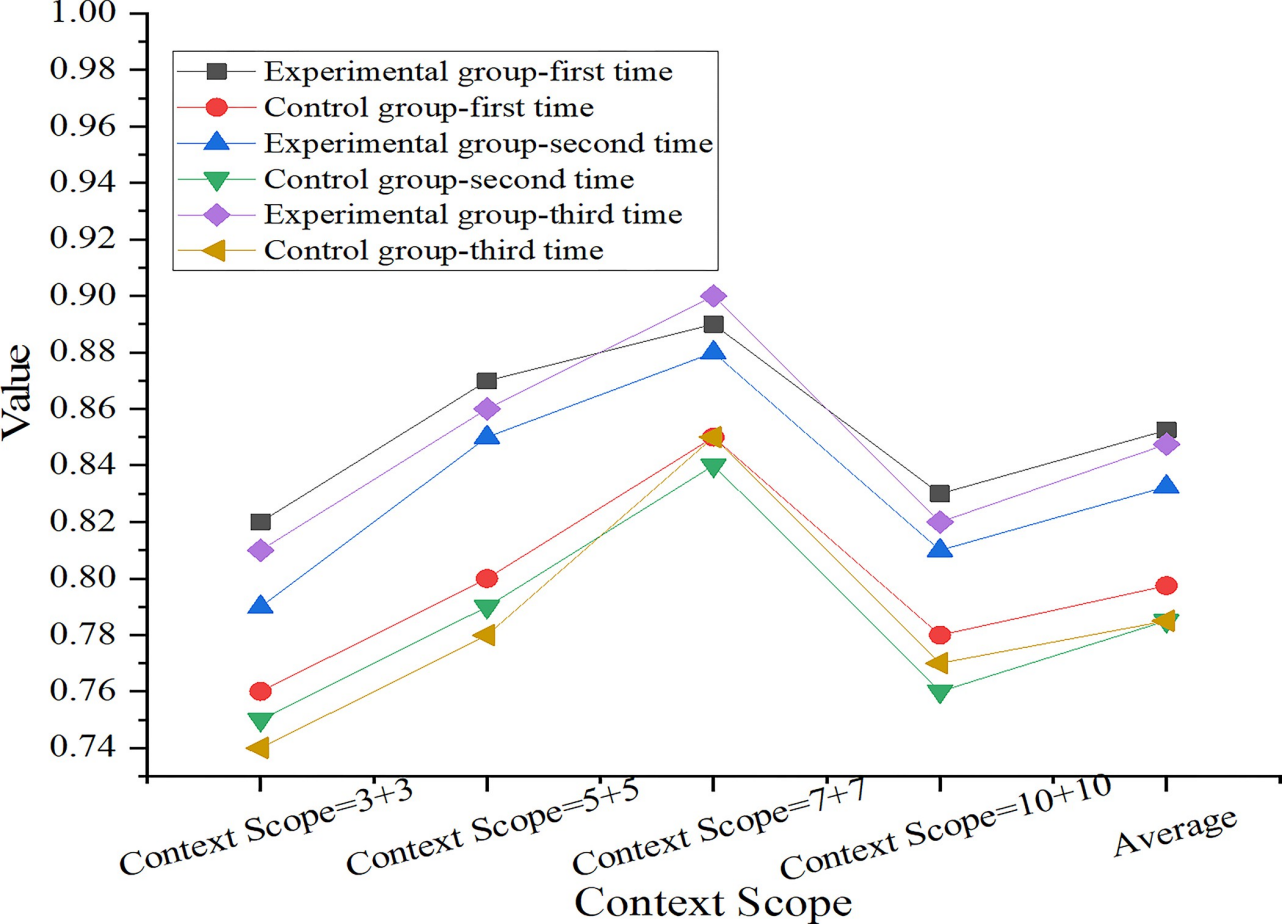
Comparison chart of syntactic analysis effect and result.

The diagram is drawn according to Table 5, as shown in Fig 4.

According to the data in Fig 4, compared with the control group, the accuracy of syntactic analysis in the experimental group reaches 89.5%, while that in the control group is only 73.2%. With the increase of sample size, the accuracy of the experimental group and the control group fluctuated, but the overall trend showed good stability. Because of the combination of SMOSS model and improved LSTM model, a comprehensive syntactic analysis framework is formed, which makes full use of their advantages and improves the effect of syntactic analysis.

### Analysis of experimental results of English text error detection

In this paper, the traditional SMOSS-based text error detection method and the improved SMOSS-LSTM are used to carry out the comparative experiment of English text error detection, and the self-built data set is used to carry out the experiment for three times. The results are shown in Table 6.

**Table 6:** Experimental results of English text error detection.

Number of experiments	Group	50	100	150
**1**	Comprehensive analysis framework	0.930	0.942	0.948
	Text error detection based on SMOSS	0.661	0.651	0.683
**2**	Comprehensive analysis framework	0.922	0.930	0.944
	Text error detection based on SMOSS	0.633	0.651	0.667
**3**	Comprehensive analysis framework	0.911	0.931	0.947
	Text error detection based on SMOSS	0.600	0.632	0.681

**Fig 5:**
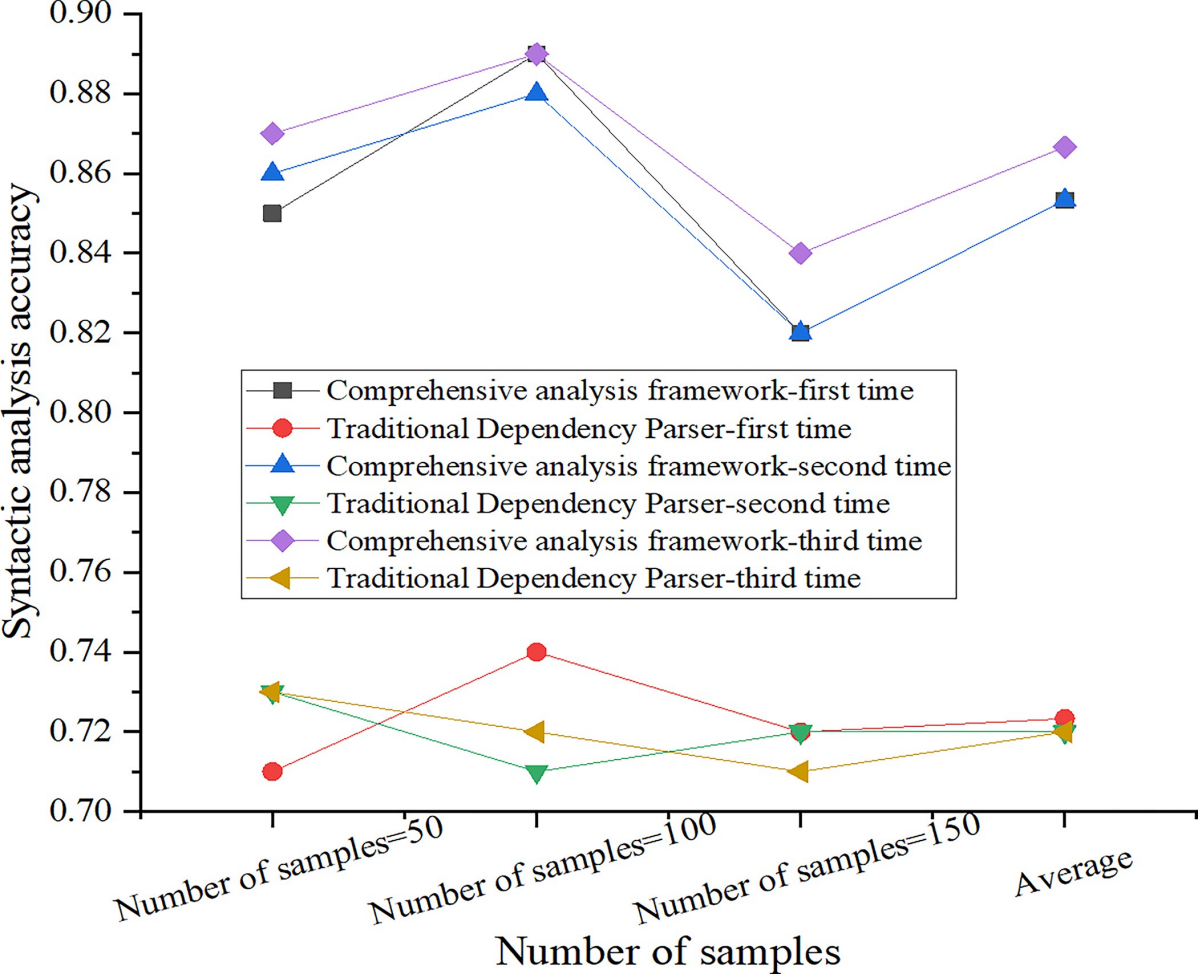
Comparison chart of English text error detection results.

The diagram is drawn according to Table 6, as shown in Fig 5.

In Fig 5, in the aspect of English text error detection, the detection accuracy of the proposed model in the experimental group is as high as 94.8%, which is significantly better than the traditional SMOSS-based text error detection method, and its accuracy is only 68.3%. In the experiment of English text error detection, the proposed SMOSS-LSTM model has a high detection accuracy in the experimental group, which is significantly better than the traditional SMOSS-based text error detection method. It also shows that with the increase of sample size, the accuracy of the experimental group tends to be stable, while the accuracy of the control group changes little. This further verifies the stability and effectiveness of SMOSS-LSTM model in dealing with complex sentences and long texts.

### Comparison with existing methods

Some popular pre-training language models, BERT, Llama (for different versions of the model, such as llama2 and llama3, the latest version is selected as far as possible, and evaluated and compared on the same task and dataset), are selected as the control group, and the "10+10" group is used in the word sense disambiguation experiment. The effect of syntactic analysis and the size of English text error detection samples are chosen as 150. The comparison results are shown in Table 7.

**Table 7:** The result compared with other existing methods.

Model	Accuracy of word sense disambiguation effect	Accuracy of syntactic analysis effect	Accuracy of English text error detection effect
The model in this paper	0.831	0.840	0.948
BERT	0.853	0.875	0.935
Llama	0.821	0.867	0.925

According to the table data, this model is slightly inferior to BERT in word sense disambiguation, slightly superior to Llama. Additionally, this model is slightly inferior to BERT in syntactic analysis but superior to BERT in English text error detection. Specifically, the accuracy of word sense disambiguation, syntactic analysis and English text error detection is 0.831, 0.840 and 0.948, respectively. In contrast, BERT’s word sense disambiguation effect is slightly better than this model, which is 0.853, but its syntactic analysis and English text error detection are slightly lower than this model, which are 0.875 and 0.935 respectively. Llama is close to this model in word sense disambiguation and English text error detection, but slightly lower than this model in syntactic analysis. Therefore, on the whole, although it is slightly inferior to BERT in word sense disambiguation, this model performs well in syntactic analysis and English text error detection, and its comprehensive performance is excellent.

## Discussion

From the experimental results, whether the sample size is 50, 100 or 150, the accuracy of the experimental group (using SMOSS-LSTM model) remains at a high level, and the highest is 94.8%. This shows that the proposed SMOSS-LSTM model has obvious advantages in English text error detection, and verifies its application potential in English writing teaching. In contrast, the control group of the traditional text error detection method based on SMOSS has a low accuracy, and the highest accuracy is only 68.3%. This further verifies the significant improvement of the proposed model compared with the traditional method. From the experimental results, it is also observed that the accuracy of the experimental group changes little under different sample sizes, which shows that the proposed SMOSS-LSTM model is stable when dealing with data of different sizes. This shows that the model has good adaptability to English texts with different lengths and complexity, and can be applied to a variety of practical scenarios, including English writing teaching for learners, natural language processing and other fields. The experimental results also show that the accuracy of the experimental group tends to be stable with the increase of sample size. This phenomenon may be related to the characteristics of LSTM model, which has advantages in dealing with long-term dependence. Compared with other existing methods, this result is due to the characteristics of each model when dealing with different types of tasks. BERT may be more effective than this model in dealing with word sense disambiguation, because it is based on Transformer architecture and can capture richer semantic information and contextual relationships. However, for the tasks of syntactic analysis and English text error detection, this model adopts a comprehensive framework combining SMOSS model and improved LSTM model, which makes full use of context information and grammatical structure, so it performs well in these tasks. In addition, Llama may be close to this model in word sense disambiguation and English text error detection, but slightly lower in syntactic analysis, which may be related to its model design and feature extraction methods.

## Conclusions

In this paper, the method of combining SMOSS model with improved LSTM model is explored and applied to the syntactic analysis task in English writing teaching. The experimental results verify the effectiveness and advantages of the proposed SMOSS-LSTM model in English text error detection. By comparing the results of the experimental group and the control group, it is observed that the accuracy of the experimental group is significantly higher than that of the control group. The experimental results show that with the increase of sample size, the accuracy of the experimental group tends to be stable, which shows that the proposed SMOSS-LSTM model is stable when dealing with data of different sizes, and has the advantage of adapting to different text lengths and complexity. However, the proposed model also has some limitations. Firstly, the sample size of experimental data is relatively small, and the diversity of samples is insufficient, which may limit the generalization ability of the model in wider application scenarios. Secondly, the model may encounter performance bottlenecks when dealing with very complex grammatical structures or extremely long texts, especially when computing resources are limited. In addition, although SMOSS-LSTM model performs well in specific tasks, its applicability in other NLP tasks has not been fully verified. The future research direction should focus on expanding the scale and diversity of datasets to enhance the reliability of experimental results and the generalization ability of the model. Meanwhile, the model can be applied to other natural language processing tasks, such as text classification and machine translation, to evaluate its applicability and universality. In addition, the model structure is further optimized to improve its computational efficiency, so that it can better meet the practical application needs in real life, especially in the resource-limited environment.

## Supporting information

S1 Data The relevant data in this manuscript can be found in this file. (ZIP)
