# Supplementary figures and images for: Syntactic analysis of SMOSS model combined with improved LSTM model: Taking English writing teaching as an example
Source: PLoS One. 2024 Nov 15;19(11):e0312049. doi: 10.1371/journal.pone.0312049 (PMC11567549; doi:10.1371/journal.pone.0312049)

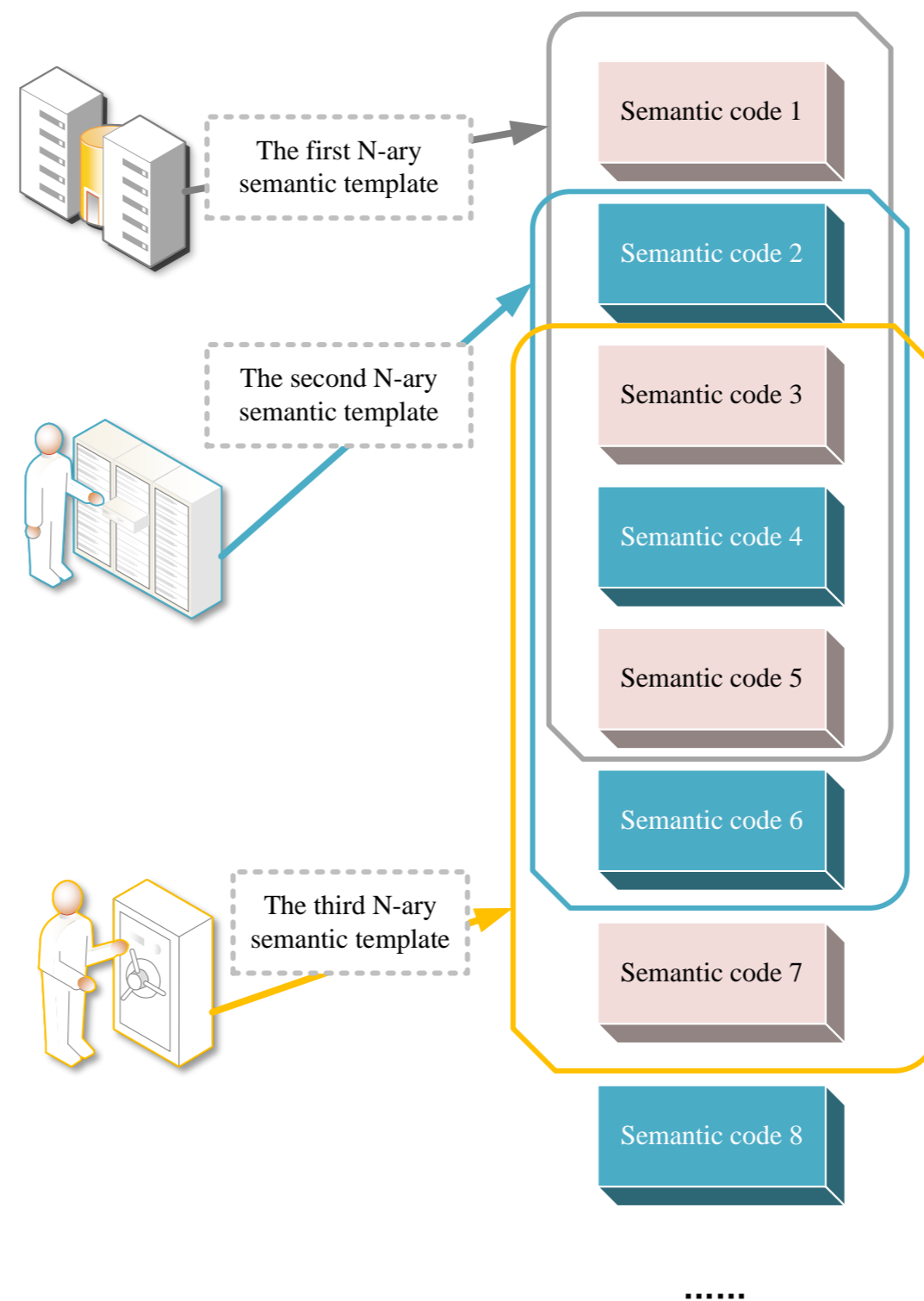

Supplement: S1 Data — (ZIP) [file pone.0312049.s001.zip › Data/Framework and model chart/Figure 1.pdf]

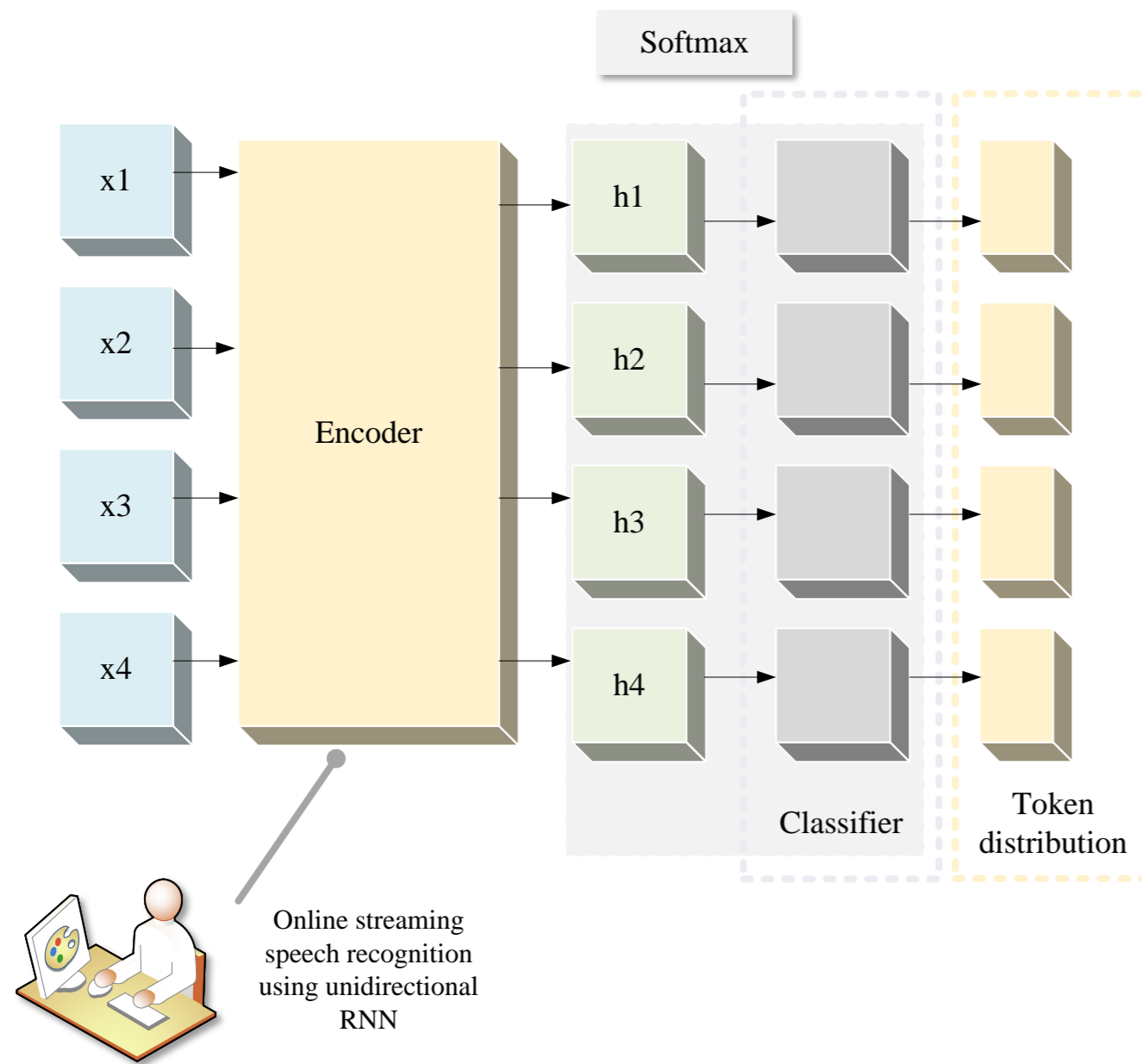

Supplement: S1 Data — (ZIP) [file pone.0312049.s001.zip › Data/Framework and model chart/Figure 2.pdf]
